# Identification of therapeutic targets and prognostic biomarkers in cholangiocarcinoma *via* WGCNA

**DOI:** 10.3389/fonc.2022.977992

**Published:** 2022-12-14

**Authors:** Lei Xu, Ting Xiao, Ling Xu, Wei Yao

**Affiliations:** ^1^ Department of Pediatrics Affiliated Tongji Hospital, Tongji Medical College, Huazhong University of Science and Technology, Wuhan, Hubei, China; ^2^ Department of Ultrasonography Affiliated Tongji Hospital, Tongji Medical College, Huazhong University of Science and Technology, Wuhan, Hubei, China; ^3^ Department of Nursing Affiliated Tongji Hospital, Tongji Medical College, Huazhong University of Science and Technology, Wuhan, Hubei, China; ^4^ Department of Oncology Affiliated Tongji Hospital, Tongji Medical College, Huazhong University of Science and Technology, Wuhan, Hubei, China

**Keywords:** cholangiocarcinoma, weighted gene co-expression network analysis, therapeutic target, prognostic signature, bioinformatics & computational biology

## Abstract

**Background:**

Cholangiocarcinoma (CCA) is a highly aggressive malignant tumor for which limited treatment methods and prognostic signatures are available. This study aims to identify potential therapeutic targets and prognostic biomarkers for CCA.

**Methods:**

Based on differentially expressed genes (DEGs) identified from The Cancer Genome Atlas (TCGA) data, our study identified key gene modules correlated with CCA patient survival by weighted gene coexpression network analysis (WGCNA). Cox regression analysis identified survival-related genes in the key gene modules. The biological properties of the survival-related genes were evaluated by CCK-8 and transwell assays. Then, these genes were used to construct a prognostic signature that was internally and externally validated. Additionally, by combining clinical characteristics with the gene−based prognostic signature, a nomogram for survival prediction was built.

**Results:**

WGCNA divided the 1531 DEGs into four gene modules, and the yellow gene module was significantly associated with overall survival (OS) and histologic neoplasm grade. Our study identified the lncRNA *AGAP2−AS1* and a novel gene, *GOLGA7B*, that are closely related to survival. *GOLGA7B* downregulation promoted the invasion, migration and proliferation of CCA cells, but *AGAP2−AS1* had the opposite effect. *AGAP2−AS1* and *GOLGA7B* were integrated into a gene−based prognostic signature, and both internal and external validation studies confirmed that this two-gene prognostic signature and nomogram could accurately predict CCA patient prognosis.

**Conclusion:**

*AGAP2−AS1* and *GOLGA7B* are potential therapeutic targets and prognostic biomarkers for CCA.

## Introduction

Cholangiocarcinoma (CCA) is a highly aggressive malignancy of the biliary duct tree. It can be classified into distal CCA (dCCA), perihilar CCA (pCCA), and intrahepatic CCA (iCCA) (1, 2). CCA is the second most common primary hepatic carcinoma, comprising approximately 15% of all primary liver tumors and 3% of gastrointestinal carcinomas (1, 3, 4). Although CCA is rare, its morbidity rate (0.3-6 per 100,000 people) and mortality rate (1–6 per 100,000 people) have increased in recent decades (4–6). In addition, due to the lack of symptoms of early-stage disease and the scarcity of efficient early diagnostic methods, CCA patients are usually diagnosed in advanced stages (7). Less than one-third of CCA patients are eligible to undergo radical surgery (8). Even in cases that receive radical surgery, chemotherapy, and radiation therapy, the risk of recurrence and metastasis is high, so the 5-year survival rate is low, and the prognosis is dismal (8, 9). Thus, it is imperative to conduct an in-depth investigation of the molecular mechanism of CCA development and identify novel biomarkers for early diagnosis and prognosis prediction.

Over the past two decades, RNA sequencing has undergone significant progress and has become an indispensable tool for carcinoma research, contributing to biomarker discovery and the development of cancer mRNA vaccines, targeted therapy, and immunotherapy; moreover, it has significantly improved the understanding of therapeutic and prognostic strategies for some malignancies, such as non-small cell lung carcinoma, leukemia and melanoma (10–13). Based on RNA sequencing, various studies have identified differentially expressed genes (DEGs), including prognostic biomarkers and signal transduction pathways, involved in CCA progression (12, 14). However, the application of relevant research to clinical practice guidelines is still very limited. In addition, although some novel treatments, such as targeted therapy and immunotherapy, have been explored in clinical trials, those trials were limited and presented some unsatisfactory results (1, 15, 16). Thus, it is still necessary to elucidate the precise molecular processes of CCA and identify effective treatment targets and putative prognostic biomarkers.

In this study, the mRNA expression profiles of CCA patients were downloaded from The Cancer Genome Atlas (TCGA) and analyzed to identify DEGs. Weighted gene coexpression network analysis (WGCNA) was used to identify key gene modules associated with CCA prognosis based on the DEGs. Then, univariate and multivariate Cox regression analyses identified *GOLGA7B* and *AGAP2-AS1*, which are related to survival, from the key gene module. We confirmed that *GOLGA7B* and *AGAP2−AS1* are closely related to CCA progression *in vitro* and are potential therapeutic targets. Moreover, *GOLGA7B* and *AGAP2−AS1* were used to create a two-gene prognostic signature for CCA prognosis prediction. Both internal and external validation confirmed that the two-gene prognostic signature could accurately predict CCA patient prognosis. Finally, a nomogram combining the two-gene signature with certain clinical characteristics was built and verified to improve the prognostic prediction capacity for CCA patients.

## Materials and methods

### Dataset collection

The mRNA expression patterns (level 3) and corresponding clinical features of 36 CCA patients in the training group were obtained from the TCGA database and downloaded from UCSC Xena. In addition, the mRNA expression profiles and clinical traits of 30 CCA patients in the testing group were collected from the Gene Expression Omnibus (GEO) database (accession: GSE107943) (17).

### Differential gene expression analysis

DEGs between tumor and normal tissues were determined from the mRNA expression profiles through the “edgeR” R package (version 3.36.0) (18). Benjamini-Hochberg multiple testing correction was performed to determine the false discovery rate (FDR). We set FDR<0.05 and |log2-fold change (FC)|>1 as the thresholds for DEG identification.

### Coexpression module construction

The DEGs in the training group were employed to build the gene coexpression modules, which were established by the WGCNA method based on the “WGCNA” R package (version: 1.70-3) (19, 20). Compared to similar methods, WGCNA is superior for analyzing gene association patterns and correlating gene coexpression modules with clinical features. The construction of coexpression modules based on the WGCNA method included the following main steps. First, a correlation coefficient matrix between genes, called an adjacency matrix, was built. To ensure a scale-free network within the adjacency matrix, an appropriate soft thresholding power *β* (range of 0–30) and correlation coefficient threshold were chosen. Second, a topological overlap matrix (TOM) was constructed based on the adjacency matrix. Third, hierarchical clustering for TOM-based dissimilarity (dissTOM) was executed to obtain the hierarchical clustering tree from which the dynamic tree cut method identified the gene coexpression modules. The minimum number of genes in each gene coexpression module was set as 30, and the cut height threshold for merging similar gene modules was set as 0.25. Finally, Pearson’s correlation analysis was performed to verify the correlation between gene coexpression modules and clinical parameters; thus, the key gene module most significantly associated with clinical parameters was determined for subsequent analysis.

### Functional enrichment analyses

Kyoto Encyclopedia of Genes and Genomes (KEGG) and Gene Ontology (GO) enrichment analyses were performed to evaluate the biological activities and signaling pathways of genes in the gene coexpression modules based on the “clusterProfiler” (version 4.2.2), “org.Hs.eg.db” (version 3.14.0), “enrichplot” (version 1.14.2) and “ggplot2” (version 3.3.5) packages.

### Prognostic signature construction

With the “survival” R package (version: 3.3-1), univariate and multivariate Cox regression analyses were performed to identify the independent prognostic genes significantly related to patient survival from the key gene modules. Then, these survival-related genes were used to construct a prognostic signature for CCA patients, and each CCA patient was assigned a risk score (RS), which was calculated by the following formula:


$$RS=\sum_{k=1}^{n}Coef_{k}\times exp_{k}$$

In the formula, “*n*” indicates the number of independent prognostic genes, “*Coef_k_*” signifies the multivariate Cox regression coefficient index of gene k, and “*exp_k_*” symbolizes the mRNA expression level of gene k in the prognostic signature. The RS was calculated for each patient in the training group. CCA patients were categorized as high- and low-risk groups with the median RS as the cutoff, with patients in the high-risk group having an RS above the median value.

### Gene−based prognostic signature validation

Further, we internally validated the predictive value of the gene−based prognostic signature in the training group from TCGA and externally validated it in a testing group from GEO. First, Kaplan-Meier survival curves were generated to compare the survival rates between the high- and low-risk groups based on the “survival” (version: 3.3-1) and “survminer” (version: 0.4.9) R packages. Second, the area under the receiver operating characteristic (ROC) curve (AUC) for survival prediction was calculated by the “survivalROC” R package (version: 1.0.3). Third, the risk plot was used to visualize the differences in survival between the high- and low-risk groups and was displayed by the “heatmap” R package (version: 1.0.12). Finally, the predictive prognostic value of the gene−based prognostic signature was estimated by the concordance index (C-index) based on the “survcomp” R package (version: 1.44.1) (21).

### Nomogram construction and validation

Nomograms are widely used and are convenient devices for survival prediction in cancer patients in oncology research (22, 23). By combining the gene−based prognostic signature with relevant clinical characteristics, our study constructed a nomogram with the “rms” (version: 6.3-0), “foreign” (version: 0.8-82) and “survival” (version: 3.3-1) R packages. The C-index and the AUC of the time-dependent ROC curve were calculated to estimate the predictive prognostic value of the nomogram. In addition, calibration curves for 1-, 3- and 5-year overall survival (OS) were generated to assess the accuracy of the predicted probability.

### Patients and clinical sample collection

Paired CCA tumor and normal tissues were collected from 9 patients who did not receive chemotherapy or radiation at Tongji Hospital, Tongji Medical College, Huazhong University of Science and Technology, China, between March 2019 and January 2022. The clinical information of the nine patients is shown in **Table S1**. This study was authorized by the Tongji Hospital Research Ethics Committee and the Institutional Review Board.

### Cell culture and transfection

CCA cell lines were cultured in RPMI-1640 medium (Gibco, CA) containing 10% fetal bovine serum (FBS) at 37°C in a 5% CO_2_ atmosphere. *GOLGA7B* and *AGAP2−AS1* were knocked down by small interfering RNAs (siRNAs) using siRNA transfection reagent (Santa Cruz Biotechnology Inc., USA). The sequences of the siRNAs are shown in **Table 1**.

**Table 1:** The siRNA for target genes.

Gene	Name	SS sequence	AS sequence
GOLGA7B	siRNA-GOLGA7B -1	GGUAAGUGUUCCUGAUCAACA	UUGAUCAGGAACACUUACCUG
	siRNA-GOLGA7B -2	GGUGUUUAAGCAAGUUUAAGU	UUAAACUUGCUUAAACACCGG
	siRNA-GOLGA7B -3	GGUUCCUAGUAGAUAUCAAGG	UUGAUAUCUACUAGGAACCUA
AGAP2-AS1	siRNA-AGAP2-AS1 -1	GAGCAAUCCGAGUGUGGAAAC	UUCCACACUCGGAUUGCUCUG
	siRNA-AGAP2-AS1 -2	GACACGAUCAGGAACUCAAAG	UUGAGUUCCUGAUCGUGUCCA
	siRNA-AGAP2-AS1 -3	CCACUUGUUACCUGCUUUAUA	UAAAGCAGGUAACAAGUGGGG

### Western blotting and quantitative real-time PCR

The protein expression of *GOLGA7B* was detected by Western blotting analysis according to the standard protocol, and the primary and secondary antibodies are listed in **Table 2**. Quantitative real-time PCR (qRT-PCR) was employed to detect RNA expression levels. TRIzol reagent (Life Technologies, CA) was used to separate total RNA from tissue samples or cell lines, and a reverse transcriptase kit (Takara Bio Inc., Dalian, China) was used to convert that RNA into complementary DNA (cDNA). qRT-PCR was conducted using the SYBR Premix EX Taq Kit (Takara Bio Inc.) according to the standard protocol. The primers for qRT-PCR listed in **Table 2** were acquired from Primer Bank. The protein expression of GOLGA7B was detected by Western blotting analysis according to the standard protocol, and the primary and secondary antibodies are listed in **Table 3**.

**Table 2:** The primers for target genes.

Gene	Forward primer	Reverse primer
GOLGA7B	TCCTGCTGTCTTCGCTACCTGAG	GGGCATCATTGGCTGGACATCTC
AGAP2-AS1	TCTGCTCTCCTCTCACACGACTTC	CCACCCTCTGCTTTCCCTACCC
B-ACTIN	CAGATGTGGATCAGCAAGCAGGAG	AAGCCATGCCAATGAGACTGAGAAG

**Table 3:** The antibody information of the target gene.

Gene	Primary antibody	Secondary antibody
GOLGA7B	NBP1-56754 (Novus)	Goat anti-rabbit IgG H&L (HRP) (abs20002), 1/3000 dilution
B-ACTIN	Abs119600 (Absin)	Goat anti-rabbit IgG H&L (HRP) (abs20002), 1/3000 dilution

### Transwell invasion and cell proliferation assays

Cell invasion was evaluated by a transwell invasion assay. In brief, 1 × 10^5^ cells/well were seeded in the upper transwell chamber containing 200 μl of serum-free RPMI-1640 culture medium. The lower chamber was supplied with 500 µl of RPMI-1640 culture medium containing 20% FBS. After 24 h of culture, the cells that had migrated through the membranes were fixed with methanol, stained with 1% crystal violet and counted by Image-Pro Plus version 6.0 (Media Cybernetics Inc., MD, USA). Cell proliferation was evaluated by CCK-8 (Dojindo Laboratories Co., Ltd., Kumamoto, Japan). In brief, 5× 10^3^ cells/well were plated in 96-well plates and cultured at 37°C for 24 h, 48 h, 72 h and 96 h. After CCK-8 solution was added to each well and incubated for 2 h, the cell proliferation was assessed according to the OD value at 450 nm, as measured by a Quant ELISA Reader (BioTek Instruments, VT, USA).

## Results

### Identification of DEGs and gene coexpression modules

**Figure 1:**
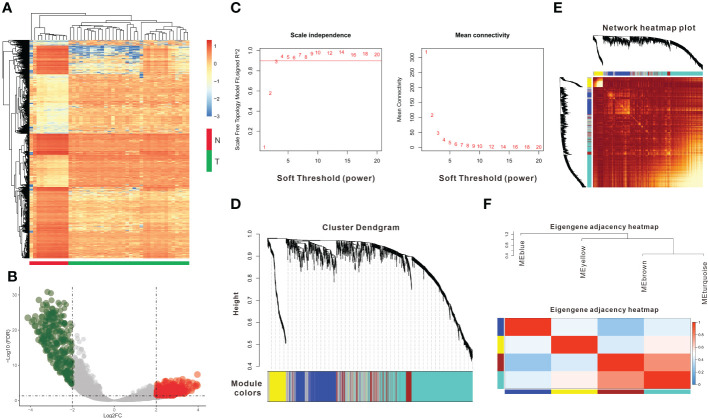
Identification of DEGs and coexpression modules. **(A)** The heatmap plot shows DEGs between tumor and normal tissues in CCA. **(B)** The volcano plot shows 849 genes downregulated and 682 genes upregulated in tumor tissues. **(C)** Determination of the soft thresholding power (β) for a correlation coefficient of 0.9, as shown by the red line in the left panel. **(D)** Clustering dendrograms show that four coexpression modules could be constructed based on all DEGs. **(E)** Network heatmap plot of the four modules; light red indicates a more significant correlation between genes, whereas darker red indicates a weaker correlation. **(F)** The eigengene adjacency heatmap shows the correlations between the 4 modules.DEGs: differentially expressed genes.

A total of 36 CCA patients from the TCGA dataset were used as the training group. DEG analysis between normal (n = 9) and tumor (n = 36) tissues was conducted, and a total of 1531 DEGs were identified (|log2 FC|>1 and FDR<0.05) (**Figure 1A** and **Table S2**). Tumor tissues overexpressed 682 genes and downregulated 849 genes compared with normal tissues (**Figure 1B**). Then, we used the 1531 DEGs to construct gene coexpression modules based on the WGCNA method. After 3 was chosen as the optimal soft thresholding power β to ensure that the network was scale-free (**Figure 1C**), the hierarchical clustering tree showed four gene coexpression modules and 323 oligogenes (**Figure 1D**). The four gene coexpression modules were the turquoise module with 637 genes, the brown module with 149 genes, the blue module with 294 genes, and the yellow module with 128 genes; the gray module included 323 oligogenes that were not classified into any module (**Figure 1D**). The correlations between the DEGs and each of the four modules were visualized with dissTOM (**Figure 1E**). The correlations among the 4 modules are shown in **Figure 1F**.

### Determination of key modules related to clinical features

**Figure 2:**
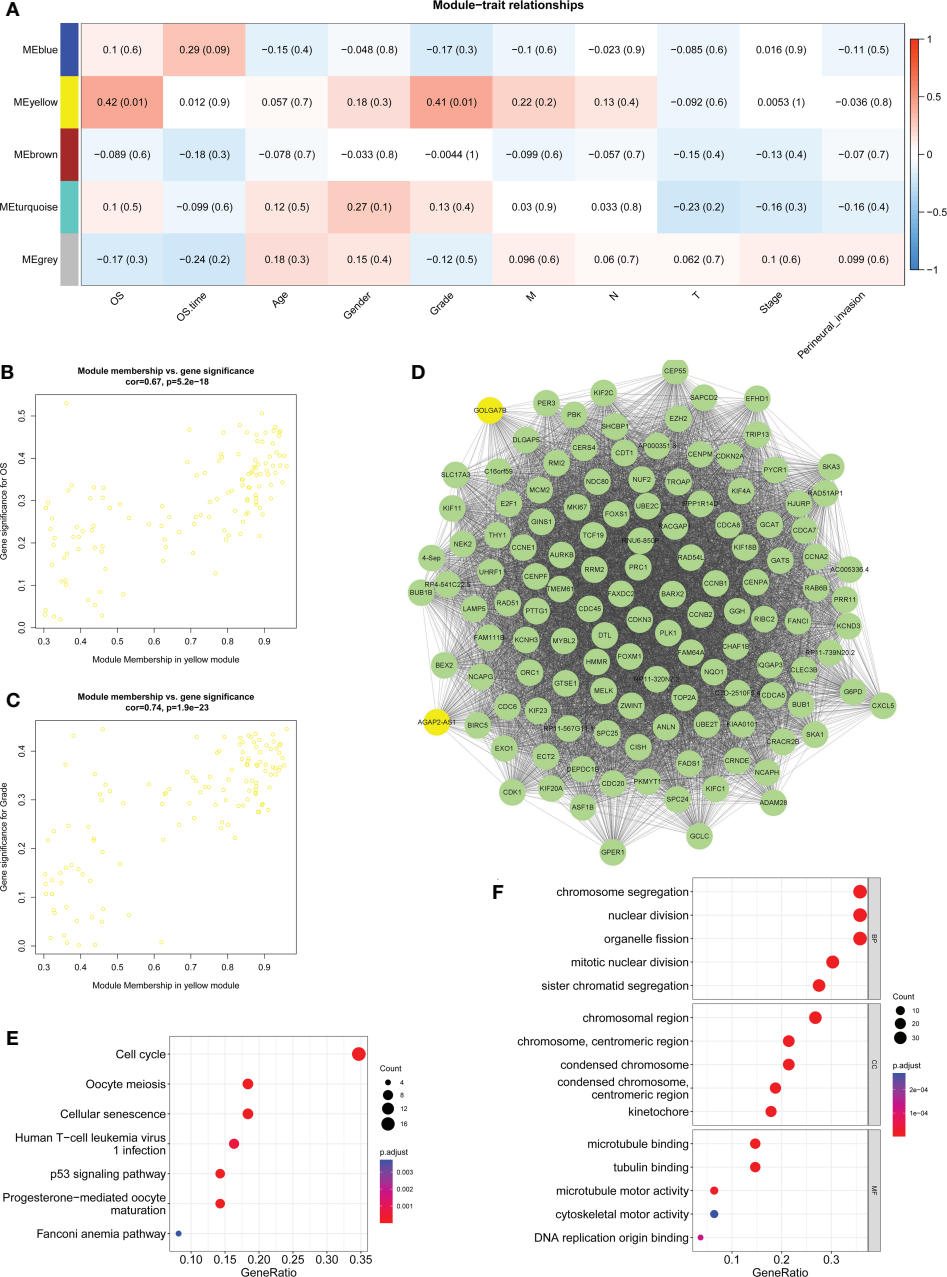
Determination of key modules related to clinical features. **(A)** Heatmap displaying the correlation between the coexpression modules and clinical traits and highlighting the yellow module as significantly related to survival and histologic neoplasm grade. **(B)** Scatter plot showing the correlation between gene significance and survival in the yellow module. **(C)** Scatter plot showing the correction between the gene significance and histologic neoplasm grade in the yellow module. **(D)** Gene interaction network of the 128 genes in the yellow module. **(E)** KEGG pathway enrichment results for genes from the yellow module. **(F)** GO-BP, GO-CC and GO-MF enrichment analyses of genes from the yellow module. BP, biological process; CC, cellular component; MF, molecular function.

The correlations between the gene coexpression modules and clinical features in the training group were calculated and are shown in **Figure 2A**. As a result, the yellow module was significantly related to survival (R = 0.42, *P* = 0.01) and neoplasm histologic grade (R = 0.41, *P* = 0.01) (**Figure 2A**). The genes in the yellow module had a highly positive relationship with OS (**Figure 2B**, R = 0.67, *P* = 5.2e-18) and neoplasm histologic grade (**Figure 2C**, R = 0.74, *P* =1.9e-23). The 128 genes from the yellow module were used to construct the gene interaction network shown in **Figure 2D**. Then, KEGG and GO enrichment analyses were performed for the 128 genes to investigate the biological processes involved. KEGG pathway enrichment analyses indicated that the cell cycle, oocyte meiosis, the p53 signaling pathway, cellular senescence, progesterone-mediated oocyte maturation, human T-cell leukemia virus 1 infection, and the Fanconi anemia pathway were enriched in the yellow module (**Figure 2E**). GO enrichment analyses indicated that chromosome segregation, nuclear division, sister chromatid segregation, mitotic unclear division and organelle fission were the top 5 biological processes; chromosomal region, chromosome (centromeric region), condensed chromosome (centromeric region), condensed chromosome, and kinetochore were the top 5 cellular components; microtubule binding, tubulin binding, microtubule motor activity, DNA replication origin binding, and cytoskeletal motor activity were the top 5 molecular functions (**Figure 2F**).

### Identification of key genes related to survival

**Figure 3:**
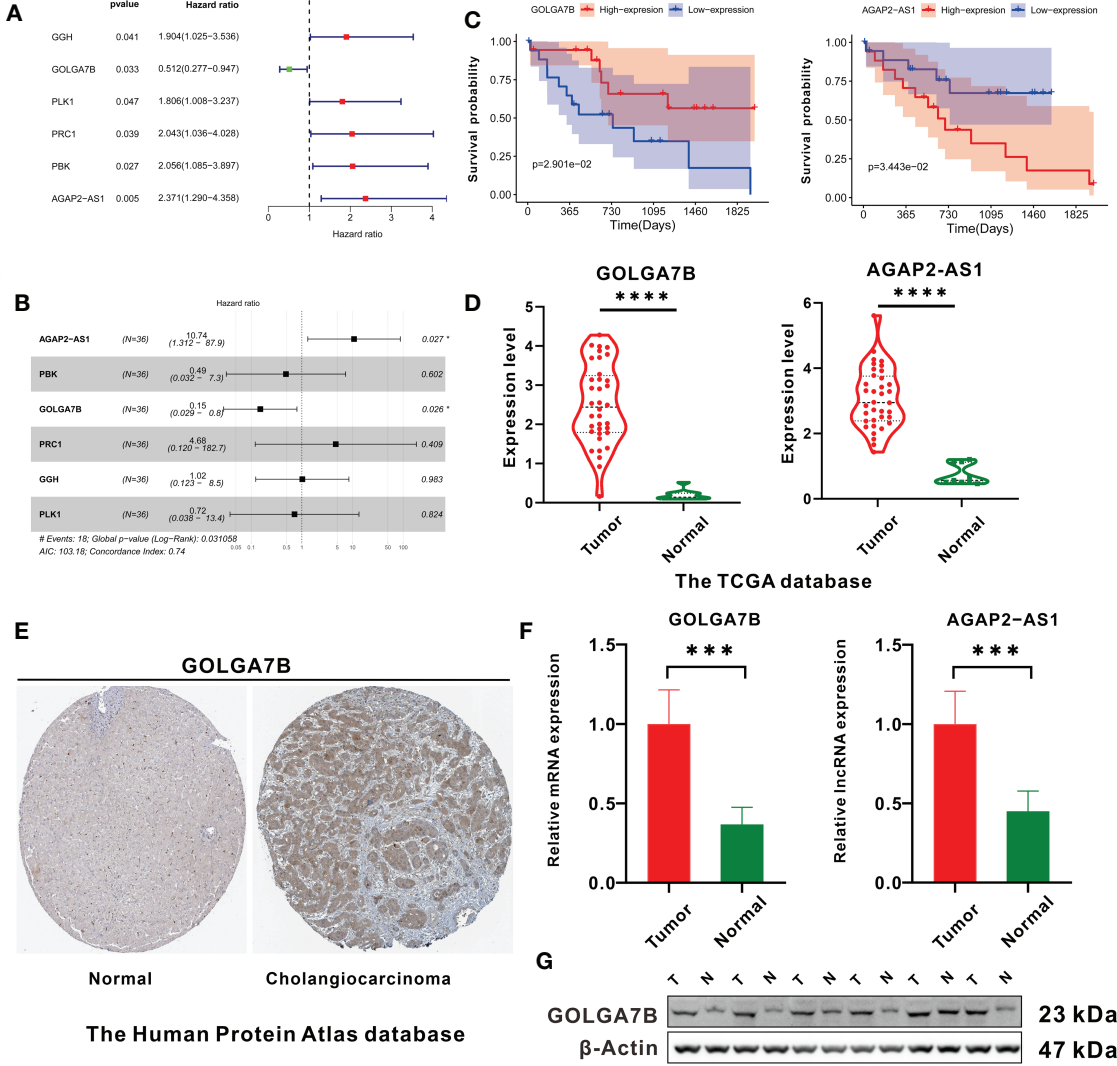
Identification of key genes related to CCA patient survival. **(A)** The forest plot shows the six survival-related genes in the yellow module identified by univariate Cox regression analysis. **(B)** Forest plot showing that *GOLGA7B* and *AGAP2−AS1* were independent survival-related genes determined through multivariate Cox regression analysis. **(C)** Kaplan–Meier curves showing that CCA patients with higher expression of *GOLGA7B* had a high survival probability, and those with a higher expression of *AGAP2−AS1* had a lower survival probability. **(D)** mRNA expression of *GOLGA7B* and *AGAP2−AS1* between tumor and normal tissues in CCA patients in the training group. **(E)** Analysis of Human Protein Atlas database indicated that GOLGA7B protein expression was significantly upregulated in CCA tumor tissues compared with cholangiocytes. The CCA tumor tissue came from a 67-year-old man (patient ID: 3334; staining: medium; quantity: 25%-75%; intensity: moderate). The normal tissue came from a 55-year-old man (patient ID: 2399; staining: not detected; quantity:<25%; intensity: weak). **(F)** qRT-PCR confirmed that *GOLGA7B* and *AGAP2−AS1* were overexpressed in tumor samples compared to normal tissue samples. **(G)** Western blotting confirmed that the protein expression of GOLGA7B was significantly upregulated in tumor samples compared with normal tissue samples. * *P*<0.05; *** *P*<0.001; **** *P*<0.0001.

Univariate Cox regression analysis was performed and verified that *GOLGA7B, AGAP2−AS1, PBK, PRC1, PLK1* and *GGH* were significantly related to survival (**Figure 3A**). Then, multivariate Cox regression analysis was performed and identified the genes *GOLGA7B* and *AGAP2−AS1* as significantly associated with survival (**Figure 3B**). High expression of *GOLGA7B* was associated with increased survival probability, while high expression of *AGAP2−AS1* was associated with decreased survival probability (**Figure 3C**).

Compared with normal tissues, both *GOLGA7B* and *AGAP2−AS1* RNA were highly expressed in tumor tissues (**Figure 3D**). In addition, the Human Protein Atlas database confirmed that the protein expression of *GOLGA7B* was significantly higher in CCA tumor tissues (**Figure 3E**). Moreover, Western blotting and qRT-PCR experiments were performed on nine pairs of matched CCA normal and tumor tissues and indicated that the protein and mRNA expression levels of *GOLGA7B* were significantly higher in tumor tissues than in normal tissues (**Figure 3F, G**) and that the lncRNA *AGAP2−AS1* was substantially expressed at lower levels in normal tissues than in tumor tissues (**Figure 3F**).

### Identification of the biological functions of *GOLGA7B* and *AGAP2−AS1*

**Figure 4:**
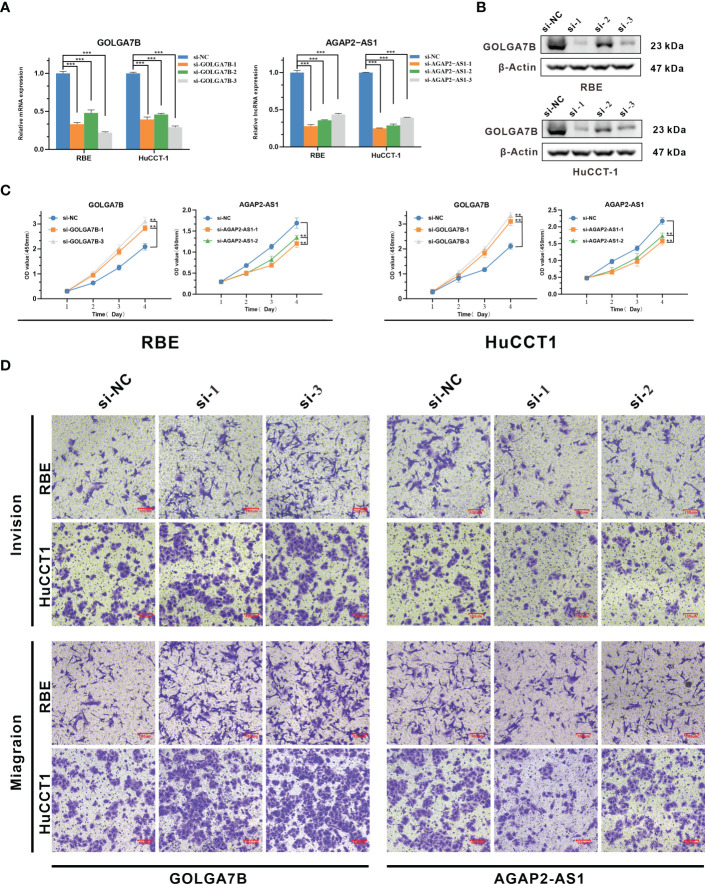
Identification of the biological functions of *GOLGA7B* and *AGAP2−AS1* in CCA. **(A)** Knockdown of *GOLGA7B* and *AGAP2−AS1* in RBE and HuCCT-1 cells was confirmed at the mRNA level by qRT-PCR. **(B)** Knockdown of GOLGA7B in RBE and HuCCT-1 cells was confirmed at the protein level by Western blotting. **(C)** The CCK-8 assay verified the proliferation of RBE and HuCCT-1 cells after *GOLGA7B* and *AGAP2−AS1* depletion. **(D)** Transwell assays were performed to measure the migration and invasion capabilities of RBE and HuCCT-1 cells after *GOLGA7B* and *AGAP2−AS1* depletion. ** *P*<0.01; *** *P*<0.001.

As the functional enrichment analyses above showed that the genes in the yellow module were mainly enriched in cell proliferation, we further confirmed the biological functions of *GOLGA7B* and *AGAP2−AS1 in vitro*. *GOLGA7B* and *AGAP2−AS1* were knocked down in both RBE and HuCCT-1 cells by *GOLGA7B* and *AGAP2−AS1* siRNAs, respectively, as confirmed by qRT-PCR (**Figure 4A**) and Western blotting experiments (**Figure 4B**). The CCK-8 assay confirmed that *GOLGA7B* downregulation increased proliferation (**Figure 4C**), and the transwell assay confirmed that *GOLGA7B* downregulation promoted cell migration and invasion (**Figure 4D**), while *AGAP2−AS1* downregulation had the opposite effect on cell growth, migration, and invasion (**Figure 4C, D**).

### Construction and internal validation of the two-gene prognostic signature

**Figure 5:**
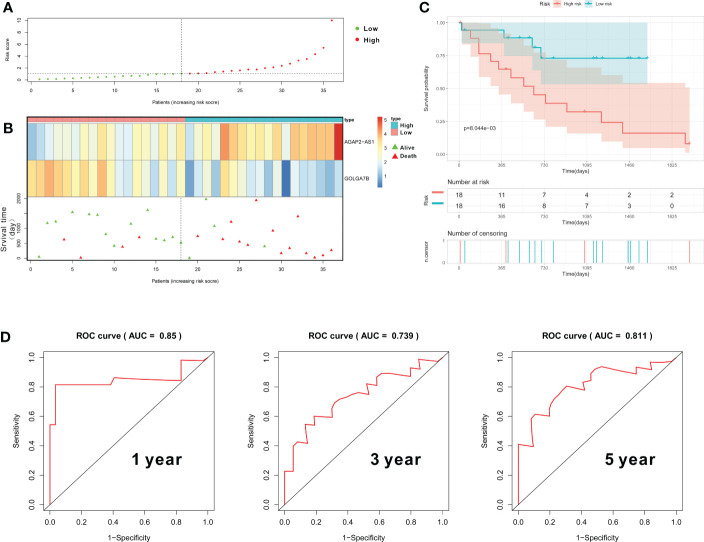
Construction and validation of the gene prognostic signature in the training group. **(A)** The risk plot shows the RS of each CCA patient. **(B)** The risk plot shows that CCA patients with higher RS values exhibited lower expression of *GOLGA7B*, higher expression of *AGAP2−AS1*, and a better survival rate. **(C)** Kaplan–Meier curves confirmed that CCA patients in the high-risk group had a poorer survival probability. **(D)** ROC curve to evaluate the diagnostic competence of the two-gene prognostic predictive signature based on the expression of *GOLGA7B* and *AGAP2−AS1*.

In our study, we further constructed a prognostic gene signature based on *GOLGA7B* and *AGAP2−AS1*, and each patient was assigned an RS that was calculated based on the expression values of these two genes and their corresponding multivariate Cox regression analysis coefficients (**Table S3**). The formula used to calculate the RS is presented above in the Materials and Methods section. We further conducted internal validation to verify the predictive value of the RS based on the training cohort’s two-gene signature. The median RS was used to categorize all CCA patients into high- and low-risk groups, with patients in the high-risk group having an RS above the median value (**Table S3**). The RSs of all 36 patients were presented in a risk plot (**Figure 5A**). The expression of *AGAP2−AS1* increased gradually with increasing RS, while the expression of *GOLGA7B* decreased with increasing RS (**Figure 5B**). The high-risk group mainly comprised nonsurviving patients (**Figure 5B**). Kaplan-Meier survival analysis verified that CCA patients with a high RS had a worse OS than those with a low RS (**Figure 5C**). In addition, the AUC of the ROC curve was calculated to verify the diagnostic competence of the two-gene prognostic signature, and the AUCs of the two-gene prognostic signature for the 1-year, 3-year, and 5-year survival predictions were 0.85, 0.739, and 0.811, respectively (**Figure 5D**). The C-index for the internal validation group was 0.734.

### External validation of the two-gene prognostic signature

**Figure 6:**
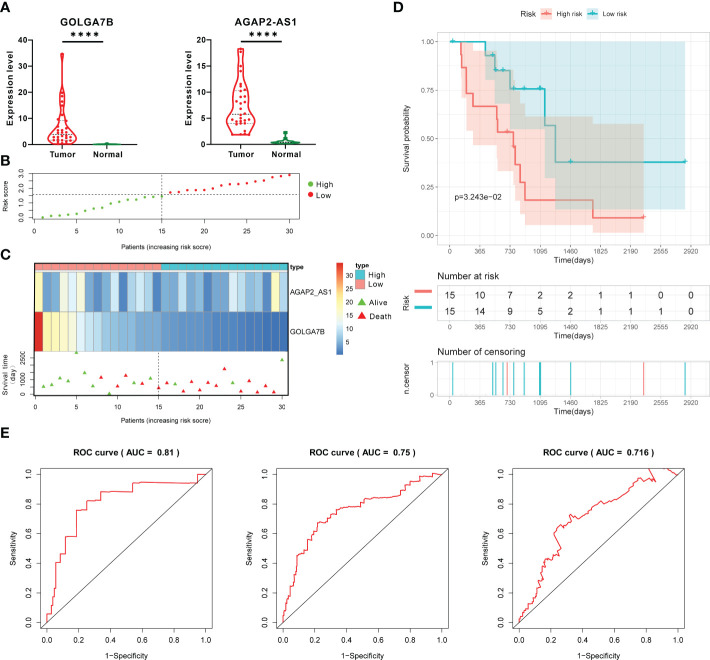
External validation of the prognostic gene signature in the testing group. **(A)** The RNA expression of *GOLGA7B* and *AGAP2−AS1* in tumor tissues was higher than that in normal tissues of CCA patients in the testing group. **(B)** The risk plot shows the RS of each CCA patient in the testing group. **(C)** The risk plot shows that CCA patients with higher RSs were associated with lower GOLGA7B expression and a better survival rate. **(D)** Kaplan–Meier curves confirmed that in the testing group, CCA patients in the high-risk group had a lower survival probability. **(E)** ROC curve and AUC values for validating the predictive competence of the two-gene prognostic prediction signature. **** *P*<0.0001.

To determine whether the two-gene prognostic signature could reliably predict prognosis in various populations, a total of 30 CCA patients from the GEO database were designated as the testing group. GOLGA7B and AGAP2−AS1 were highly expressed in tumor tissues from the testing group, similar to the case in the training group (**Figure 6A**). On the basis of the Cox proportional hazards model, the RS of each patient was also calculated according to the RNA expression level of each gene and its regression coefficient, as described above. Thirty CCA patients were then classified into high- and low-risk groups with the median RS value as the cutoff (**Table S4**). A risk plot showing the RSs of the 30 patients was constructed (**Figure 6B**). The risk plot showed that the expression of *GOLGA7B* decreased with increasing RS (**Figure 6C**). The surviving patients were mainly assigned to the low-risk group, and the nonsurviving patients were mainly assigned to the high-risk group (**Figure 6C**). Kaplan-Meier survival analysis verified that CCA patients with a low RS had a better probability of OS (**Figure 6D**). In addition, the AUCs of the ROC curves for the 1-year, 3-year, and 5-year survival predictions were 0.81, 0.75, and 0.716, respectively (**Figure 6E**). The C-index for the external validation group was 0.714.

### The landscape of tumor-infiltrating immunocytes in CCA

**Figure 7:**
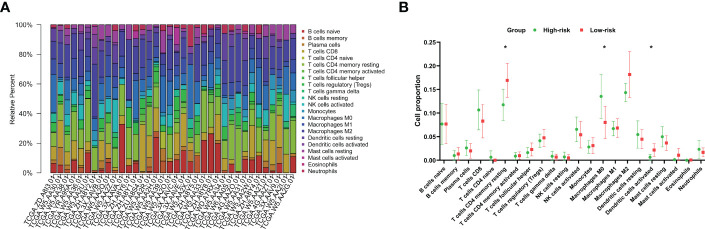
Characteristics of immunocytes in CCA. **(A)** CIBERSORT was used to calculate the relative percentages of 22 types of immunocytes for each CCA patient in the training cohort. **(B)** The mean relative proportions of the high- and low-risk groups. *P<0.05.

As the presence of tumor-infiltrating immunocytes is closely related to tumor prognosis (24), we performed CIBERSORT analysis to calculate the proportions of 22 types of tumor-infiltrating immunocytes for each CCA patient in the training group (**Figure 7A**) (25–27). Our research demonstrated that patients in the high-risk group had significantly lower levels of activated dendritic cells (P=0.035) and resting memory CD4 T cells (P=0.031) than those in the low-risk group but significantly higher levels of M0 macrophages (P=0.05) (**Figure 7B**).

### Construction of the prognostic signature combining clinical parameters with the gene signature

**Figure 8:**
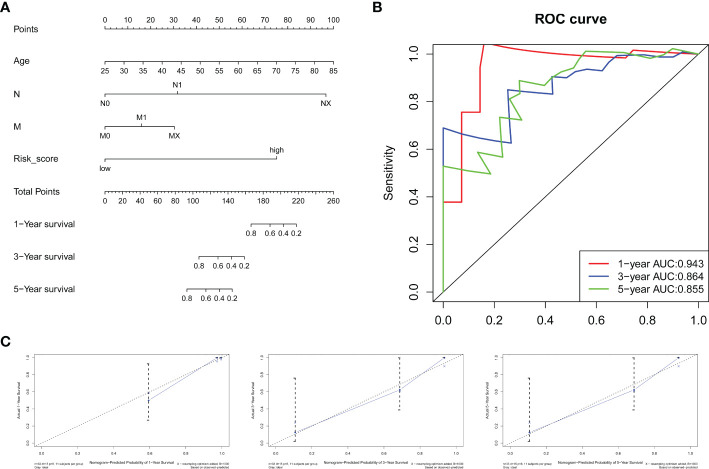
Nomogram for survival prediction. **(A)** A nomogram was built by combining the RS and clinical characteristics. **(B)** AUCs of ROC curves for 1-, 3-, and 5-year survival predictions were used to estimate the predictive accuracy of the nomogram. **(C)** Calibration plots predicting the 1-, 3- and 5-year survival probabilities of CCA patients. The predicted survival probability is displayed on the x-axis, and the actual survival probability is presented on the y-axis.

Using the training group, we combined the RS with clinical information including sex, age, grade, TNM stage and perineural invasion to construct a prognostic nomogram. Three patients without perineural invasion information were excluded. The Cox proportional hazards model was applied to evaluate the independent prognostic ability of the signature. Then, age (*P* = 0.0139), N stage (*P*=0.0231), RS (*P*=0.0243) and M stage (*P*=0.0799), which have prognostic value for OS in the training group, were incorporated into the nomogram (**Figure 8A**). The AUCs of the ROC curves for the 1-year, 3-year, and 5-year survival predictions of the nomogram were 0.943, 0.864 and 0.855, respectively, indicating a powerful capacity to differentiate patients with a favorable prognosis from patients with a poor prognosis (**Figure 8B**). In addition, the calibration curve showed excellent consistency between the predicted and observed survival probabilities at 1, 3 and 5 years (**Figure 8C**). Moreover, the C-index for the training group was 0.8597. These results revealed that the nomogram could accurately predict CCA patient survival.

## Discussion

CCA is a highly lethal and challenging to treat malignancy that is characterized by late diagnosis, early recurrence and metastasis, low resectability, frequent development of drug resistance and poor prognosis (2, 15, 28). In clinical practice, novel therapies such as immunotherapy and targeted therapy improve prognosis and significantly benefit patients with some cancers, including lung carcinoma, leukemia, melanoma and hepatocellular carcinoma (29–32). The use of these novel therapies for CCA was tested in clinical trials and showed some promising results, but the improvement of long-term prognosis was limited (2, 33, 34). To identify more useful therapeutic targets and potential biomarkers for the treatment and prognosis prediction of CCA patients, deeper and more thorough insights into the molecular processes of CCA progression are still needed (35, 36). RNA sequencing is a powerful tool for carcinoma research, significantly improving the understanding of the transcriptome characteristics of malignancies and providing potential prognostic biomarkers and therapeutic targets (10, 11, 37). Our study aimed to uncover novel predictive biomarkers and therapeutic targets for CCA by bioinformatic analysis based on the CCA mRNA transcriptome and related clinical data.

We identified 1531 DEGs in 36 CCA patients from the TCGA dataset. Then, we used the WGCNA method to divide the DEGs into four gene coexpression modules (the turquoise module with 637 genes, the brown module with 149 genes, the blue module with 294 genes, and the yellow module with 128 genes) and confirmed that the yellow module was significantly associated with CCA patient survival. WGCNA is an algorithm that can obtain gene coexpression modules from mRNA expression profiles, and genes within the same module are considered functionally related. In addition, WGCNA can identify the key modules that are significantly associated with clinical characteristics (19, 38).

In our study, WGCNA confirmed that the yellow module was significantly related to histologic neoplasm grade, in addition to survival. Cancers with high histologic grade are characterized by poor differentiation, rapid division and proliferation, and increased invasion and metastasis (39). Moreover, GO and KEGG pathway enrichment analyses indicated that the genes in the yellow module were mainly enriched in the biological processes chromosome segregation and nuclear division and the cell cycle pathway. In summary, enrichment analyses revealed that the genes in the yellow module were closely associated with cell division and proliferation. Thus, the key gene module defined and confirmed by WGCNA was reliably associated with clinical characteristics, and the results of WGCNA have biological significance and could be used for further analysis (14).

To detect the key genes that could serve as prognostic biomarkers and therapeutic targets, we performed univariate and multivariate Cox regression analyses to identify survival-related genes in the yellow module. We verified that the *GOLGA7B* and *AGAP2−AS1* genes were significantly related to the survival of CCA patients. Upregulated *GOLGA7B* was a protective factor related to good prognosis, while *AGAP2−AS1* was a risk factor associated with poor prognosis.

GOLGA7B is a novel accessory protein that has rarely been studied in the context of carcinoma. Woodley et al. showed that S-acylated GOLGA7B could interact with and stabilize DHHC5 at the plasma membrane to enhance cell adhesion and restrain cell scatter (40). Loss of cell adhesion causes loss of contact inhibition, which results in increased cell migration, cell proliferation and cancer progression (41, 42). We also found that downregulated *GOLGA7B* led to enhanced proliferation, invasion and metastasis of CCA cells. Meanwhile, *AGAP2−AS1* is a lncRNA and was recognized as an oncogene in several types of cancer, such as adenocarcinoma, laryngeal squamous cell carcinoma, breast cancer and lung cancer (43–45). Some studies have elucidated the molecular process by which *AGAP2−AS1* promotes cancer progression. Pengyu et al. reported that AGAP2-AS1 promoted cell invasion and proliferation by upregulating the miR-193a-3p/LOXL4 pathway in laryngeal squamous cell carcinoma (45). Yan et al. confirmed that AGAP2-AS1 regulated the migration, invasion, proliferation and apoptosis of glioma cells *via* the miR-628-5p/PTN axis (46). We found that AGAP2-AS1 modulated the proliferation, migration and invasion of CCA, and Ji et al. confirmed that SP1-induced AGAP2-AS1 promoted CCA proliferation by silencing CDKN1A (47). Therefore, both *GOLGA7B* and *AGAP2−AS1* regulate CCA progression and are potential therapeutic targets for CCA. *GOLGA7B* is a novel and promising target, and further study is required to illustrate the molecular mechanisms by which it inhibits cancer progression.

Our study also verified that *GOLGA7B* and *AGAP2−AS1* could be prognostic biomarkers for CCA patients. We calculated an RS for each CCA patient in the training and testing groups based on the RNA expression of *GOLGA7B* and *AGAP2−AS1* and related regression coefficients. The CCA patients were categorized into low- and high-risk groups according to the RS value, and the high-risk group generally exhibited a poor prognosis. Both internal and external validation experiments verified that the two-gene prognostic signature could precisely predict CCA patient prognosis.

Recently, several prognostic gene signatures have been constructed for CCA (48). Wang et al. identified a signature consisting of five ferroptosis-related genes for predicting the prognosis of cholangiocarcinoma (49). Wang et al. created a thirteen-lncRNA prognostic signature for iCCA (50). Zou et al. built a metabolism-related 4-lncRNA prognostic model for iCCA (51). However, compared with these previous signatures, the two-gene prognostic signature defined in our study has some advantages. First, with confirmation by internal and external validation, our study indicated that the two-gene prognostic signature could precisely predict CCA patient prognosis and can be widely used. Second, *GOLGA7B* and *AGAP2−AS1* were filtered out from all DEGs and were significantly associated with clinical characteristics. Thus, these survival-related genes represented the genome-wide transcription profile, had high biological significance and could be used to construct reliable prognostic signatures. Third, only two genes were included in the construction of the prognostic signature in our study, making it much smaller than the signatures in previous studies. Therefore, this two-gene prognostic signature is more suitable for future clinical translation or the development of a detection kit for clinical applications.

Nevertheless, several limitations to our study should also be noted. Our study was a retrospective study, and all the data were acquired from public databases. In addition, the number of samples in both the training and testing groups may be insufficient. Thus, selection bias and information bias are inevitable, and further extensive prospective studies must be implemented to verify the results of our study. Moreover, our study preliminarily explored the biological properties of *GOLGA7B* and *AGAP2−AS1*. Nevertheless, clarifying the molecular pathways by which GOLGA7B and AGAP2-AS1 regulate cancer development and alter prognosis will require more *in vivo* and *in vitro* research.

## Conclusion

A prognostic signature comprising *AGAP2−AS1* and *GOLGA7B* could accurately predict the prognosis of CCA patients. AGAP2−AS1 and GOLGA7B were associated with the proliferation, invasion and metastasis of CCA cells. Both AGAP2−AS1 and GOLGA7B are potential therapeutic targets and prognostic biomarkers for CCA.

## Data availability statement

The original contributions presented in the study are included in the article/**Supplementary Material**. Further inquiries can be directed to the corresponding author.

## Ethics statement

The studies involving human participants were reviewed and approved by Tongji Hospital Research Ethics Committee. The patients/participants provided their written informed consent to participate in this study. Written informed consent was obtained from the individual(s) for the publication of any potentially identifiable images or data included in this article.

## Author contributions

LX conceived the idea, designed the study, analyzed data, performed most of the experiment, and wrote the manuscript. LX and TX provided help with analyzing data. WY supervised the entire project. All authors contributed to the article and approved the submitted version.

## References

[1] Banales JM Marin JJG Lamarca A Rodrigues PM Khan SA Roberts LR. Cholangiocarcinoma 2020: the next horizon in mechanisms and management. Nat Rev Gastroenterol Hepatol (2020) 17:557–88. doi: 10.1038/s41575-020-0310-z PMC7447603 32606456

[2] Brindley PJ Bachini M Ilyas SI Khan SA Loukas A Sirica AE. Cholangiocarcinoma. Nat Rev Dis Primers (2021) 7:65. doi: 10.1038/s41572-021-00300-2 34504109 PMC9246479

[3] Global Burden of Disease Cancer C Fitzmaurice C Dicker D Pain A Hamavid H Moradi-Lakeh M. The global burden of cancer 2013. JAMA Oncol (2015) 1:505–27. doi: 10.1001/jamaoncol.2015.0735 PMC4500822 26181261

[4] Banales JM Cardinale V Carpino G Marzioni M Andersen JB Invernizzi P. Expert consensus document: Cholangiocarcinoma: current knowledge and future perspectives consensus statement from the European network for the study of cholangiocarcinoma (ENS-CCA). Nat Rev Gastroenterol Hepatol (2016) 13:261–80. doi: 10.1038/nrgastro.2016.51 27095655

[5] Bertuccio P Malvezzi M Carioli G Hashim D Boffetta P El-Serag HB. Global trends in mortality from intrahepatic and extrahepatic cholangiocarcinoma. J Hepatol (2019) 71:104–14. doi: 10.1016/j.jhep.2019.03.013 30910538

[6] Khan SA Tavolari S Brandi G. Cholangiocarcinoma: Epidemiology and risk factors. Liver Int (2019) 39 Suppl 1:19–31. doi: 10.1111/liv.14095 30851228

[7] Izquierdo-Sanchez L Lamarca A La Casta A Buettner S Utpatel K Klumpen HJ. Cholangiocarcinoma landscape in Europe: Diagnostic, prognostic and therapeutic insights from the ENSCCA registry. J Hepatol (2022) 76:1109–21. doi: 10.1016/j.jhep.2021.12.010 35167909

[8] Khan SA Davidson BR Goldin RD Heaton N Karani J Pereira SP. Guidelines for the diagnosis and treatment of cholangiocarcinoma: an update. Gut (2012) 61:1657–69. doi: 10.1136/gutjnl-2011-301748 22895392

[9] Razumilava N Gores GJ. Cholangiocarcinoma. Lancet (2014) 383:2168–79. doi: 10.1016/S0140-6736(13)61903-0 PMC4069226 24581682

[10] Hong M Tao S Zhang L Diao L-T Huang X Huang S. RNA Sequencing: New technologies and applications in cancer research. J Hematol Oncol (2020) 13(1):166. doi: 10.1186/s13045-020-01005-x 33276803 PMC7716291

[11] Stark R Grzelak M Hadfield J. RNA Sequencing: The teenage years. Nat Rev Genet (2019) 20:631–56. doi: 10.1038/s41576-019-0150-2 31341269

[12] Huang X Tang T Zhang G Liang T. Identification of tumor antigens and immune subtypes of cholangiocarcinoma for mRNA vaccine development. Mol Cancer (2021) 20:50. doi: 10.1186/s12943-021-01342-6 33685460 PMC7938044

[13] Szeto GL Finley SD. Integrative approaches to cancer immunotherapy. Trends Cancer (2019) 5:400–10. doi: 10.1016/j.trecan.2019.05.010 PMC7467854 31311655

[14] Long J Huang S Bai Y Mao J Wang A Lin Y. Transcriptional landscape of cholangiocarcinoma revealed by weighted gene coexpression network analysis. Brief Bioinform (2021) 22(4):bbaa224. doi: 10.1093/bib/bbaa224 33051665

[15] Rizvi S Khan SA Hallemeier CL Kelley RK Gores GJ. Cholangiocarcinoma - evolving concepts and therapeutic strategies. Nat Rev Clin Oncol (2018) 15:95–111. doi: 10.1038/nrclinonc.2017.157 28994423 PMC5819599

[16] Charalampakis N Papageorgiou G Tsakatikas S Fioretzaki R Kole C Kykalos S. Immunotherapy for cholangiocarcinoma: a 2021 update. Immunotherapy (2021) 13:1113–34. doi: 10.2217/imt-2021-0126 34190581

[17] Montal R Sia D Montironi C Leow WQ Esteban-Fabro R Pinyol R. Molecular classification and therapeutic targets in extrahepatic cholangiocarcinoma. J Hepatol (2020) 73:315–27. doi: 10.1016/j.jhep.2020.03.008 PMC8418904 32173382

[18] Chen Y Pal B Visvader JE Smyth GK. Differential methylation analysis of reduced representation bisulfite sequencing experiments using edgeR. F1000Research (2017) 6:2055. doi: 10.12688/f1000research.13196.1 29333247 PMC5747346

[19] Langfelder P Horvath S. WGCNA: an r package for weighted correlation network analysis. BMC Bioinf (2008) 9:559. doi: 10.1186/1471-2105-9-559 PMC2631488 19114008

[20] Zhang B Horvath S. A general framework for weighted gene co-expression network analysis. Stat Appl Genet Mol Biol (2005) 4:Article17. doi: 10.2202/1544-6115.1128 16646834

[21] Longato E Vettoretti M Di Camillo B. A practical perspective on the concordance index for the evaluation and selection of prognostic time-to-event models. J BioMed Inform (2020) 108:103496. doi: 10.1016/j.jbi.2020.103496 32652236

[22] Balachandran VP Gonen M Smith JJ DeMatteo RP. Nomograms in oncology: more than meets the eye. Lancet Oncol (2015) 16:e173–80. doi: 10.1016/S1470-2045(14)71116-7 PMC4465353 25846097

[23] Iasonos A Schrag D Raj GV Panageas KS. How to build and interpret a nomogram for cancer prognosis. J Clin Oncol (2008) 26:1364–70. doi: 10.1200/JCO.2007.12.9791 18323559

[24] Goeppert B Frauenschuh L Zucknick M Stenzinger A Andrulis M Klauschen F. Prognostic impact of tumour-infiltrating immune cells on biliary tract cancer. Br J Cancer (2013) 109:2665–74. doi: 10.1038/bjc.2013.610 PMC3833207 24136146

[25] Chen B Khodadoust MS Liu CL Newman AM Alizadeh AA. Profiling tumor infiltrating immune cells with CIBERSORT. Methods Mol Biol (2018) 1711:243–59. doi: 10.1007/978-1-4939-7493-1_12 PMC5895181 29344893

[26] Newman AM Liu CL Green MR Gentles AJ Feng W Xu Y. Robust enumeration of cell subsets from tissue expression profiles. Nat Methods (2015) 12:453–7. doi: 10.1038/nmeth.3337 PMC4739640 25822800

[27] Finotello F Mayer C Plattner C Laschober G Rieder D Hackl H. Molecular and pharmacological modulators of the tumor immune contexture revealed by deconvolution of RNA-seq data. Genome Med (2019) 11:34. doi: 10.1186/s13073-019-0638-6 31126321 PMC6534875

[28] Rodrigues PM Olaizola P Paiva NA Olaizola I Agirre-Lizaso A Landa A. Pathogenesis of cholangiocarcinoma. Annu Rev Pathol (2021) 16:433–63. doi: 10.1146/annurev-pathol-030220-020455 33264573

[29] Sangro B Sarobe P Hervas-Stubbs S Melero I. Advances in immunotherapy for hepatocellular carcinoma. Nat Rev Gastroenterol Hepatol (2021) 18:525–43. doi: 10.1038/s41575-021-00438-0 PMC8042636 33850328

[30] Llovet JM Castet F Heikenwalder M Maini MK Mazzaferro V Pinato DJ. Immunotherapies for hepatocellular carcinoma. Nat Rev Clin Oncol (2022) 19:151–72. doi: 10.1038/s41571-021-00573-2 34764464

[31] Luke JJ Flaherty KT Ribas A Long GV. Targeted agents and immunotherapies: optimizing outcomes in melanoma. Nat Rev Clin Oncol (2017) 14:463–82. doi: 10.1038/nrclinonc.2017.43 28374786

[32] Devine SM Larson RA. Acute leukemia in adults: recent developments in diagnosis and treatment. CA Cancer J Clin (1994) 44:326–52. doi: 10.3322/canjclin.44.6.326 7953914

[33] Piha-Paul SA Oh DY Ueno M Malka D Chung HC Nagrial A. Efficacy and safety of pembrolizumab for the treatment of advanced biliary cancer: Results from the KEYNOTE-158 and KEYNOTE-028 studies. Int J Cancer (2020) 147:2190–8. doi: 10.1002/ijc.33013 32359091

[34] Abou-Alfa GK Macarulla T Javle MM Kelley RK Lubner SJ Adeva J. Ivosidenib in IDH1-mutant, chemotherapy-refractory cholangiocarcinoma (ClarIDHy): a multicentre, randomised, double-blind, placebo-controlled, phase 3 study. Lancet Oncol (2020) 21:796–807. doi: 10.1016/S1470-2045(20)30157-1 32416072 PMC7523268

[35] Sirica AE Gores GJ Groopman JD Selaru FM Strazzabosco M Wei Wang X. Intrahepatic cholangiocarcinoma: Continuing challenges and translational advances. Hepatology (2019) 69:1803–15. doi: 10.1002/hep.30289 PMC6433548 30251463

[36] Macias RIR Kornek M Rodrigues PM Paiva NA Castro RE Urban S. Diagnostic and prognostic biomarkers in cholangiocarcinoma. Liver Int (2019) 39 Suppl 1:108–22. doi: 10.1111/liv.14090 30843325

[37] Kuksin M Morel D Aglave M Danlos FX Marabelle A Zinovyev A. Applications of single-cell and bulk RNA sequencing in onco-immunology. Eur J Cancer (2021) 149:193–210. doi: 10.1016/j.ejca.2021.03.005 33866228

[38] Kakati T Bhattacharyya DK Barah P Kalita JK. Comparison of methods for differential Co-expression analysis for disease biomarker prediction. Comput Biol Med (2019) 113:103380. doi: 10.1016/j.compbiomed.2019.103380 31415946

[39] Nagtegaal ID Odze RD Klimstra D Paradis V Rugge M Schirmacher P. The 2019 WHO classification of tumours of the digestive system. Histopathology (2020) 76:182–8. doi: 10.1111/his.13975 PMC7003895 31433515

[40] Say YH Sio YY Heng AHS Ng YT Matta SA Pang SL. Golgin A7 family member b (GOLGA7B) is a plausible novel gene associating high glycaemic index diet with acne vulgaris. Exp Dermatol (2022) 31(8):1208–19. doi: 10.1111/exd.14575 35416335

[41] Mendonsa AM Na TY Gumbiner BM. E-cadherin in contact inhibition and cancer. Oncogene (2018) 37:4769–80. doi: 10.1038/s41388-018-0304-2 PMC6119098 29780167

[42] Pavel M Renna M Park SJ Menzies FM Ricketts T Fullgrabe J. Contact inhibition controls cell survival and proliferation *via* YAP/TAZ-autophagy axis. Nat Commun (2018) 9:2961. doi: 10.1038/s41467-018-05388-x 30054475 PMC6063886

[43] Qian X Qu H Zhang F Peng S Dou D Yang Y. Exosomal long noncoding RNA AGAP2-AS1 regulates trastuzumab resistance *via* inducing autophagy in breast cancer. Am J Cancer Res (2021) 11:1962–81. doi: 10.21203/rs.3.rs-34252/v1 PMC8167703 34094664

[44] Zhang F Sang Y Chen D Wu X Wang X Yang W. M2 macrophage-derived exosomal long non-coding RNA AGAP2-AS1 enhances radiotherapy immunity in lung cancer by reducing microRNA-296 and elevating NOTCH2. Cell Death Dis (2021) 12:467. doi: 10.1038/s41419-021-03700-0 33972506 PMC8110970

[45] Ren P Niu X Zhao R Liu J Ren W Dai H. Long non-coding RNA AGAP2-AS1 promotes cell proliferation and invasion through regulating miR-193a-3p/LOXL4 axis in laryngeal squamous cell carcinoma. Cell Cycle (2022) 21:697–707. doi: 10.1080/15384101.2021.2016197 35113007 PMC8973330

[46] Yan Y Wang Y Liu Y Chen T Zhu Y Li H. Long non-coding RNA AGAP2-AS1/miR-628-5p/PTN axis modulates proliferation, migration, invasion, and apoptosis of glioma cells. Cancer Manag Res (2020) 12:6059–68. doi: 10.2147/CMAR.S250890 PMC7398883 32801858

[47] Ji H Wang J Lu B Li J Zhou J Wang L. SP1 induced long non-coding RNA AGAP2-AS1 promotes cholangiocarcinoma proliferation *via* silencing of CDKN1A. Mol Med (2021) 27:10. doi: 10.1186/s10020-020-00222-x 33522895 PMC7852216

[48] Pan Y Shao S Sun H Zhu H Fang H. Bile-derived exosome noncoding RNAs as potential diagnostic and prognostic biomarkers for cholangiocarcinoma. Front Oncol (2022) 12:985089. doi: 10.3389/fonc.2022.985089 36091129 PMC9449313

[49] Wang Z Zhang Y Chen Y Liu S Li C Li X. Identification of a ferroptosis-related gene signature for predicting the prognosis of cholangiocarcinoma. Expert Rev Gastroenterol Hepatol (2022) 16:181–91. doi: 10.1080/17474124.2022.2029700 35026122

[50] Zhang Z Wang Z Huang Y. Identification of potential prognostic long non-coding RNA for predicting survival in intrahepatic cholangiocarcinoma. Med (Baltimore) (2020) 99:e19606. doi: 10.1097/MD.0000000000019606 PMC7220432 32221083

[51] Zou W Wang Z Wang F Li L Liu R Hu M. A metabolism-related 4-lncRNA prognostic signature and corresponding mechanisms in intrahepatic cholangiocarcinoma. BMC Cancer (2021) 21:608. doi: 10.1186/s12885-021-08322-5 34034689 PMC8152356

